# Engineering a lipoxygenase from *cyclocybe aegerita* towards long chain polyunsaturated fatty acids

**DOI:** 10.1186/s13568-021-01195-8

**Published:** 2021-03-04

**Authors:** Dominik Karrer, Martin Gand, Martin Rühl

**Affiliations:** 1 Institute of Food Chemistry and Food Biotechnology, Justus-Liebig University Giessen, Institute of Food Chemistry and Food Biotechnology, Heinrich-Buff Ring 17, Giessen, Hesse 35392 Germany; 2 Fraunhofer Institute for Molecular Biology and Applied Ecology IME Branch for Bioresources, Department of Biology and Chemistry, Justus-Liebig University Giessen, Institute of Food Chemistry and Food Biotechnology, Heinrich-Buff Ring 17, Giessen, Hesse 35392 Germany

**Keywords:** Lipoxygenase, Mutagenesis, Biocatalysis, Protein engineering, *Cyclocybe aegerita*

## Abstract

The basidiomycetous lipoxygenase Lox1 from *Cyclocybe aegerita* catalyzes the oxygenation of polyunsaturated fatty acids (PUFAs) with a high preference towards the C18-PUFA linoleic acid (C18:2 (ω-6)). In contrast, longer PUFAs, generally not present in the fungal cell such as eicosatrienoic acid (C20:3(ω-3)) and docosatrienoic acid (C22:3 (ω-3)), are converted with drastically lower activities. With site-directed mutagenesis, we were able to create two variants with enhanced activities towards longer chain PUFAs. The W330L variant showed a ~ 20 % increased specific activity towards C20:3(ω-3), while a ~ 2.5-fold increased activity against C22:3 (ω-3) was accomplished by the V581 variant.

## Introduction

Lipoxygenases (LOX) are non-heme iron dependent dioxygenases that catalyze the insertion of molecular oxygen at a (1*Z*,4*Z*)-pentadiene motif, which occurs e.g. in polyunsaturated fatty acids (PUFAs), in a regio- and stereospecific manner (An et al. 2018; Liavonchanka and Feussner 2006). In higher fungi of the phyla *Basidiomycota*, C18-PUFAs and especially linoleic acid are predominant, whereas C20-PUFAs like arachidonic acid and eicosapentaenoic acid were either found in very low amounts or in traces in fungi of the phyla *Basidiomycota*, such as in *Cyclocybe aegerita* (syn. *Agroycbe aegerita*), and *Ascomycota* like *Phellinus* sp. and *Romaria* sp. (Brodhun and Feussner 2011, Dembitsky et al. 1991, Landi et al. 2017). In general, studies on purified basidiomycetous LOX are scarce with only LOX from *Pleurotus* spp. and *Cyclocybe aegerita* described so far (Karrer and Rühl 2019; Kuribayashi et al. 2002; Leonhardt et al. 2013; Plagemann et al. 2013). For three of them it is known that they primarily produce 13-hydroperoxy-9*Z*,11*E*-octadecadienoic acid (13-HPOD) and minor levels of 9-hydroperoxy-10*E*,12*Z*-octadecadienoic acid (9-HPOD) (Karrer and Rühl 2019; Kuribayashi et al. 2002; Plagemann et al. 2013). Furthermore, basidiomycetous LOX share a high preference towards C18-PUFAs of which linoleic acid was converted with the highest preference. With increasing chain length, the activity drastically decreases (Karrer and Rühl 2019). Yet, no study focused on the question which amino acids are the determining factors of the inefficient oxygenation of C20-C22-PUFAs. This shortcoming is addressed in this study by site-directed mutagenesis of three amino acid residues located in the substrate tunnel of the Lox1 from *Cyclocybe aegerita*.

## Materials and methods

### Cloning and protein expression of CaeLOX1

The codon optimized *LOX1* gene (accession number: MW013781), whose original cDNA was commercially purchased and cloned into the plasmid pET28a (BioCat GmbH, Heidelberg, Germany). For protein expression, pET28a/Lox1 plasmid was transformed into *E. coli* BL21-Gold (DE3) by adding ~ 100 ng of plasmid-DNA to chemically competent cells. The mixture was incubated on ice for 30 min, followed by a heat shock for 45 s at 42 °C. After 5 min rest on ice, 500 µL of LB-medium was added to the competent cells with a subsequent incubation period of 30 min at 37 °C. 200 µL of the cell suspension was plated on LB-Agar, supplemented with kanamycin (50 mg L^− 1^). After incubation over night at 37°C, the obtained clones were used for protein expression. Recombinant *E. coli* cells were cultivated in autoinduction medium containing 10 g tryptone, 5 g yeast extract, supplemented with 50 mM Na_2_HPO_4_, 50 mM KH_2_PO_4_, 25 mM (NH_4_)_2_SO_4_, 0.5 % (w/v) glycerol, 0.025 % (w/v) glucose, 0.2 % (w/v) lactose and 50 mg L^− 1^ kanamycin as selection marker, at 24 °C for 16 h. Cells were harvested by centrifugation (4.000*g*, 30 min, 4 °C) and stored at -20 °C until further use.

### Protein purification

The cell pellet was thawed on ice and resuspended in lysis-buffer (50 mM phosphate, 300 mM NaCl, pH 7.5). Disruption of cells was carried out by sonification (3 cycles for 60 s each with 60 s rest in between) on ice using a sonifier (Bandelin Sonopuls, Berlin, Germany). After complete disruption, cell debris was removed by centrifugation (14.000 *g*, 30 min, 4°C). The resulting supernatant was further processed by using Ni-NTA spin columns (Qiagen, Hilden, Germany) following manufacturer instructions. The eluted protein (using 500 mM imidazole in the elution buffer) was collected and subsequently concentrated and rebuffered in 50 mM phosphate buffer (pH 7.5) by using Pall Nanosep® omega centrifugal devices (10 kDa cut off). The concentrated protein was analyzed via SDS-PAGE. Fractions with purified CaeLox1 were used for further analysis. Protein concentration was photometrically determined by using the 260/280 ratio and the specific extinction coefficient (Ɛ_280_ = 102,135 M^− 1^ cm^− 1^), calculated with the ExPASy ProtParam tool (Gasteiger et al. 2005).

### Lipoxygenase assay

LOX activity was determined by recording the formation of the conjugated double bond at 234 nm (ε = 25,000 M^− 1^ cm^− 1^) on a Nanophotometer (Implen, Munich, Germany). The reaction mixture contained 1.25 mM PUFA (Acros Organics: linoleic acid, linolenic acid, Cayman Chemicals: eicosatrienoic acid, docosatrienoic acid), 20 µL enzyme solution and 50 mM phosphate buffer, pH 7.5 to a final volume of 1 mL.

### Determination of pH- and temperature‐optimum

For the determination of the pH-optimum, three different buffers were used: 50 mM acetate buffer, pH 4.5–6.0; 50 mM phosphate buffer, pH 6.5–7.5 and 50 mM borate buffer, pH 8.0–10.0. Effects of the temperature were determined by incubating the reaction mixture at different temperatures, ranging from 4 °C – 60 °C.

### Site‐directed mutagenesis

For mutagenesis a modified QuikChange® protocol was used (Gand et al. 2016). The modified QuikChange® reaction mixture contained 1 µL pET28a-Lox1-WT (~ 50 ng/µL), 2.5 µL forward primer, 2.5 µL reverse primer (Table 1), 1 µL dNTPs, 0.5 µL Phusion-polymerase (Thermo Fisher Scientific), 10 µL 5× GC-buffer, 1 µL dimethylsulfoxide and 35 µL ddH_2_O. PCR conditions with Phusion polymerase: initial denaturation for 3 min at 98 °C, 30 s denaturation at 98 °C, 30 s annealing at 62/64/66/68 °C, elongation at 72 °C for 3:30 min, final elongation for 5 min at 72 °C. Then, 2 µL *Dpn*I (Thermo Fisher Scientific, Waltham, MA, USA) was added and the samples were incubated for 2 h at 37 °C, followed by *Dpn*I inactivation at 80 °C for 10 min. Chemically competent *E. coli* Zymo 10β (Zymo Research Europe GmbH, Freiburg, Germany) were transformed via heat shock with the PCR-products and plated out on LB-plates containing kanamycin as selection marker (50 mg L^− 1^). After incubation over night at 37 °C, the obtained colonies were picked and used to inoculate fresh LB-medium with the according selection marker. After another incubation period over night at 37 °C, the success of site-directed mutagenesis was confirmed via DNA-sequencing (Microsynth Seqlab, Göttingen, Germany).

**Table 1:** Primer sequences for site-directed mutagenesis

primer	sequence
fw_Lox1-I393F	5′-ccgagtatggttgccccgtttagttattttaaaattccgg-3′
rv_Lox1-I393F	5′-ccggaattttaaaataactaaacggggcaaccatactcgg-3′
fw_Lox1-V581A	5′-gatgatggcacaggcgccgtatctgctgag-3′
rv_Lox1-V581A	5′-ctcagcagatacggcgcctgtgccatcatc-3′
fw_Lox1-V581F	5′-gatgatggcacagtttccgtatctgctgag-3′′
rv_Lox1-V581F	5′-ctcagcagatacggaaactgtgccatcatc-3′
fw_Lox1-W330L	5′-caggtgagcgatctgacccgtcatgaactg-3′
rv_Lox1-W330L	5′-cagttcatgacgggtcagatcgctcacctg-3′

### Homology modelling

The crystal structure of the lipoxygenase from *Glycine max* (PDB: 1IK3) was used as template, sharing 43 % sequence similarity. Models were calculated with SWISS-MODEL () (Guex et al. 2009) and visualized with Chimera 1.13.1 (Pettersen et al. 2004).

### Molecular docking

Autodock Vina was used for docking analysis. For each docking run, a box was defined which covered the active site with a grid of 22 × 18 × 26 Å. All parameters were set on standard (Trott et al. 2010).

## Results

### Bioinformatic analysis and homology modelling

**Fig. 1:**
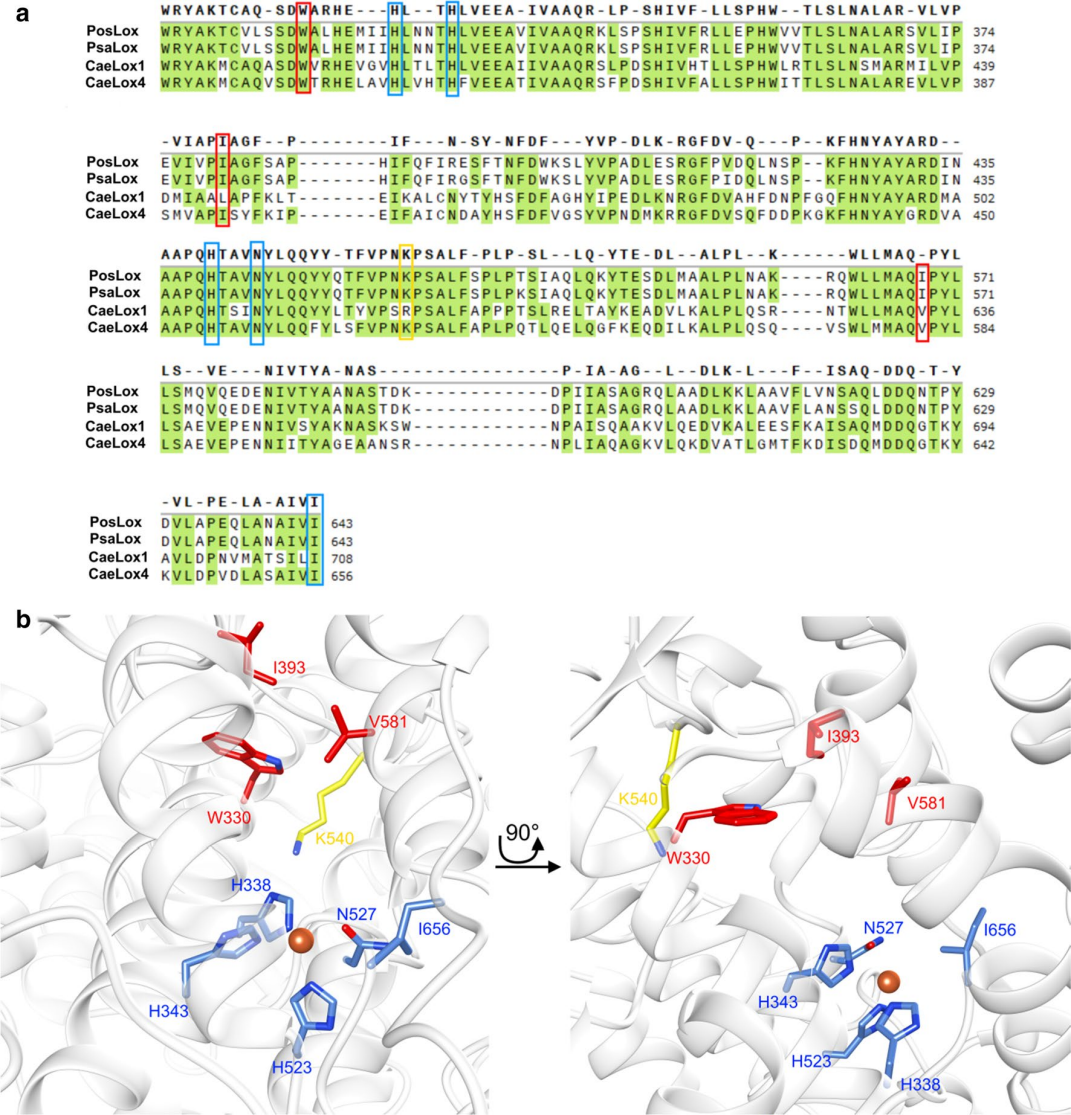
**a** Partial sequence alignment of various LOX. *Cyclocybe aegerita* CaeLox1 (MW013781), CaeLox4 (MK451709), *Pleurotus sapidus* PsaLOX (CCV01580), *Pleurotus ostreatus* PosLOX (CCV01578). Alignment was carried out by using Clustal Omega with default parameters. The highlighted amino acid residues involved in iron binding are highlighted in blue. Amino acids considered to be involved in selectivity and activity by shaping the substrate tunnel are highlighted in red. Amino acid residues considered to interact with the carboxylate end of the substrate via ionic interactions are highlighted in yellow. **b** Substrate tunnel of the homology model of CaeLox1. Iron (orange) binding amino acid residues are highlighted as blue sticks (H338, H343, H523, N527, I656) and the positively charged amino acid at the bottom of the substrate tunnel is highlighted in yellow sticks. Amino acid residues involved in shaping the substrate tunnel are shown as red sticks (V581, I393 and W330). Oxygen inside the amino acid side chains are colored in red and nitrogen in blue

**Fig. 2:**
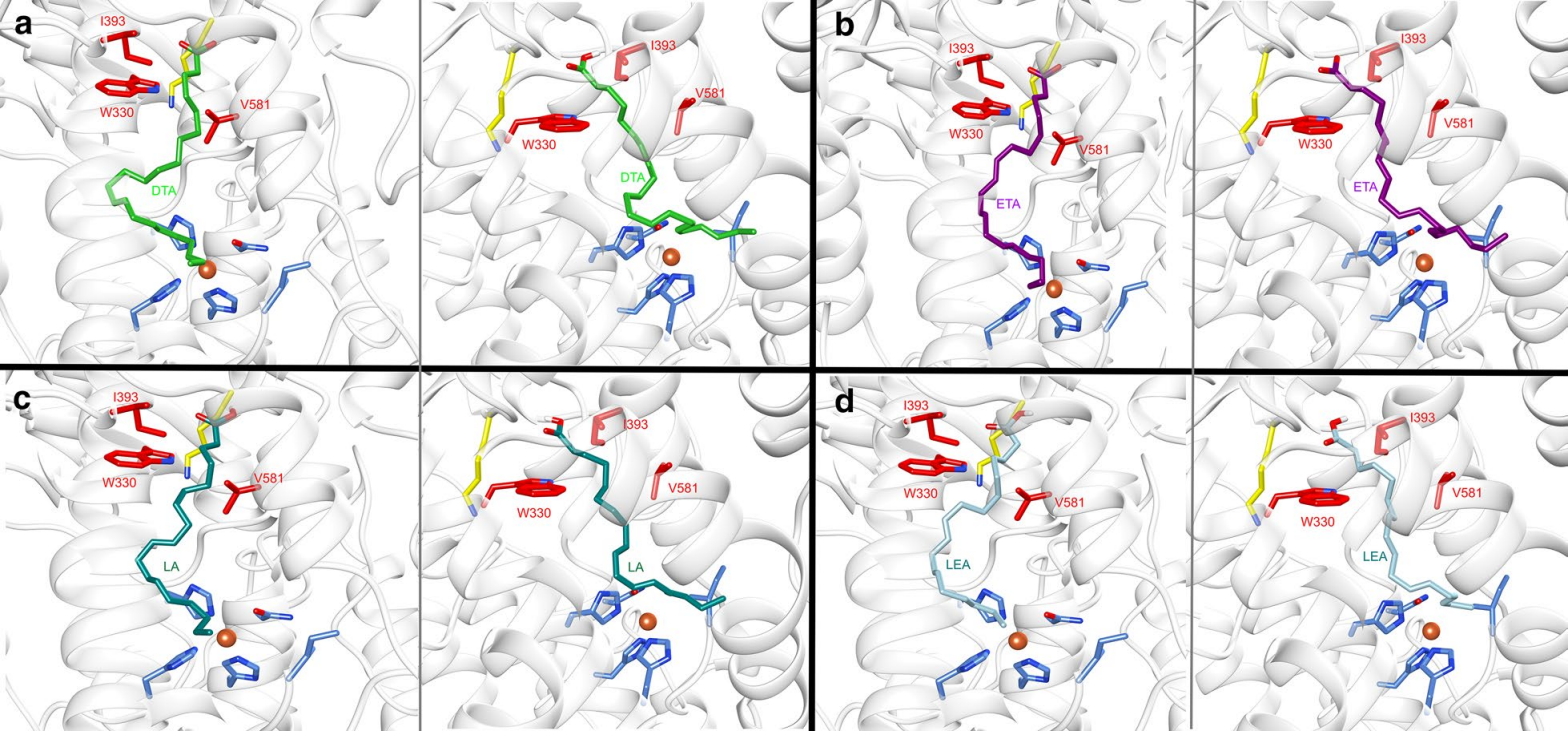
Molecular docking of **a** docosatrienoic acid (DTA, green sticks), **b** eicosatrienoic acid (ETA, magenta sticks), **c** linoleic acid (LA, cyan sticks) and **d** linolenic acid (LEA, light blue sticks) into the homology model of CaeLox1. Iron (orange) binding amino acid residues are highlighted as blue sticks (H338, H343, H523, N527, I656) and the positively charged amino acid at the bottom of the substrate tunnel is highlighted in yellow sticks. Amino acid residues involved in shaping the substrate tunnel are shown as red sticks (V581, I393 and W330). Oxygen inside the amino acid side chains are colored in red and nitrogen in blue

Shape and specificity of mammalian LOX or the regioselectivity of LOX from plants were investigated by various studies. It was pointed out that specific bulky/aliphatic amino acid residues in the substrate tunnel can affect substrate orientation and, therefore, be crucial for substrate specificity (Borngräber et al. 1999; Brodhun et al. 2013; Hornung et al. 2008). A comparison of sequence alignments of already characterized basidiomycetous LOX, assisted by the homology model of CaeLox1, shows that in their substrate tunnel the amino acid W330 is highly conserved and the amino acid hydrophobicity at the positions I393 and V581 are very similar (Fig. 1a, b). Furthermore, molecular docking in a carboxyl-end towards the stabilizing K540 orientation was calculated for the widely occurring linoleic acid (C18:2ω-6), linolenic acid (C18:3ω-3) as well as for the rare PUFAs eicosatrienoic acid (C20:3ω-3) and docosatrienoic acid (C22:3ω-3). This revealed that the residues W330, I393 and V581 are indeed in interacting distance, with less than 5 Å, to the carboxy-end and middle section of the tested PUFAs (Fig. 2a–d).

### Temperature and pH optimum

**Fig. 3:**
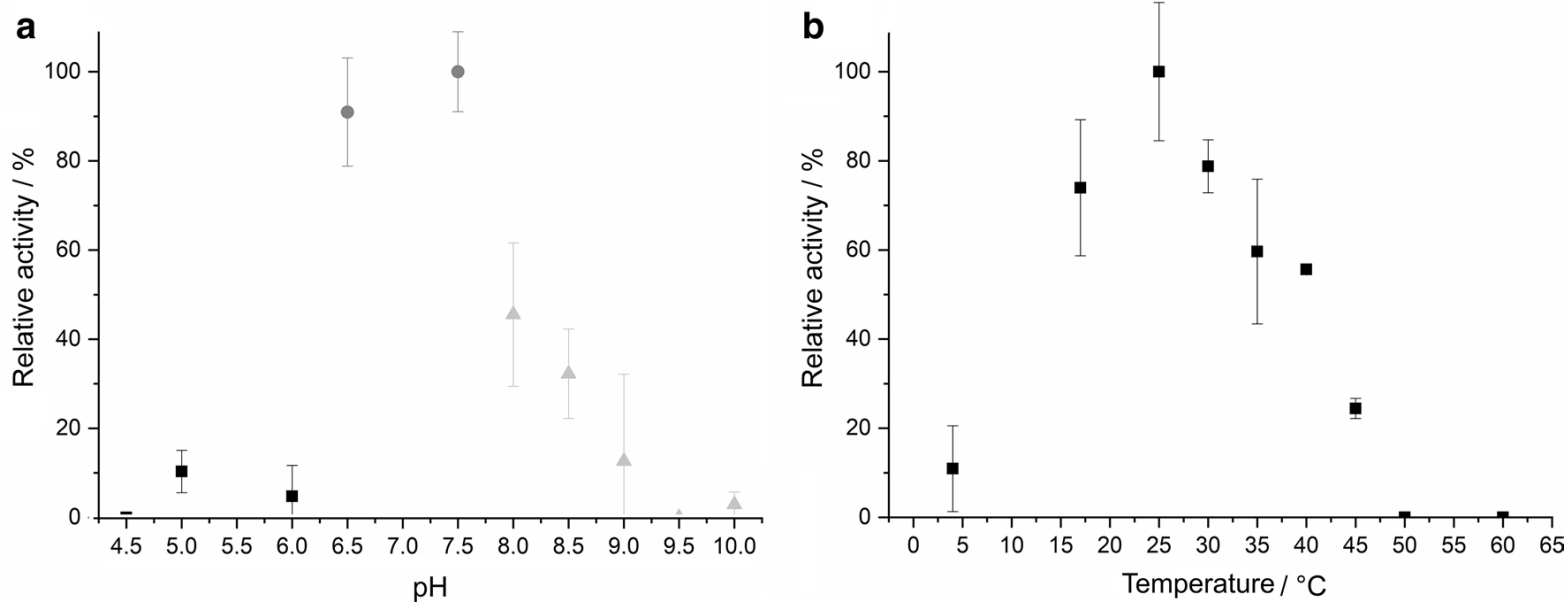
Effects of pH (**a**) and temperature (**b**) on CaeLox1 activity. buffers used: 50 mM acetate (squares) from pH 4.5–6.0, 50 mM phosphate (circles) from pH 6.5–7.5, 50 mM borate (triangles) from pH 8.0–10.0

In acidic environment (pH 4.5–6.0), the CaeLox1 activity remained very low with about 10 % of the maximum activity. A drastic increase in activity was detected with increasing pH reaching its maximum at 7.5. Further increase of the pH resulted in a drastic loss with no detectable activity from pH 9.5 and above (Fig. 3a). The effect of the temperature on the activity of CaeLox1 was determined in a temperature range between 4 ˚C and 60 ˚C. The highest activity was detected at 25°C. A steady loss of activity to about 80 % was detected when increasing the temperature from 25 to 45 °C. With a further temperature increase to 50 ˚C and 60°C no activity could be detected. At 4 ˚C 10 % of the maximal LOX activity remained (Fig. 3b). Based on these results, any further experiments were conducted at pH 7.5 and 25 °C.

### Specific activities towards various PUFAs

**Fig. 4:**
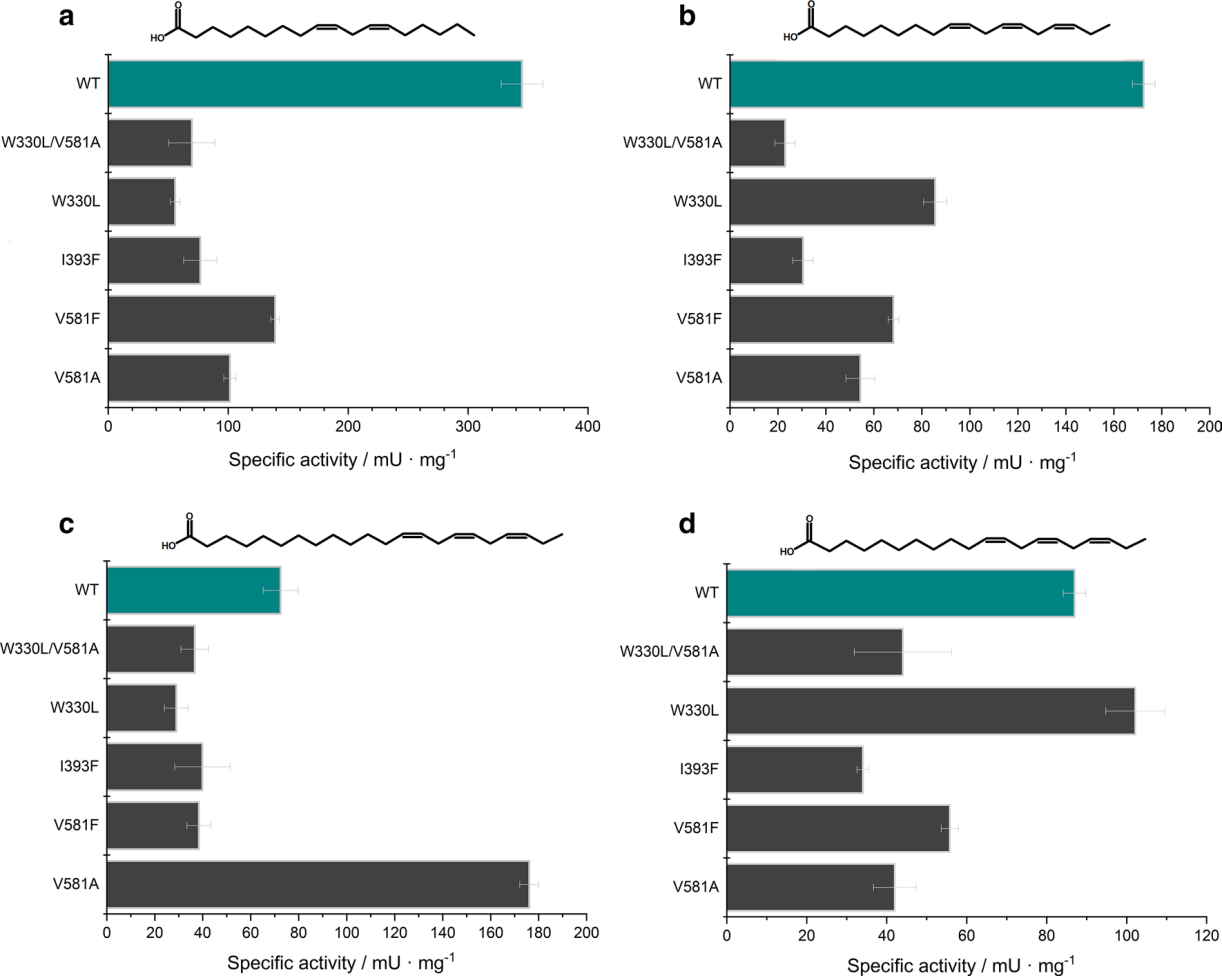
Specific activities of CaeLox1-variants compared to the wild type against **a** linoleic acid, **b** linolenic acid, **c** docosatrienoic acid and **d** eicosatrienoic acid

To experimentally verify the role of W330, I393 and V581 in PUFA oxygenation, we investigated the specific activities of PUFAs occurring (linoleic acid, linolenic acid) and non-occurring (eicosatrienoic- and docosatrienoic acid) in fungi of the phylum *Basidiomycota*. Expanding the size of the substrate tunnel (V581A), putatively stabilizing the middle-section of the substrate in a carboxylate-end first orientation (Fig. 1), led to a ~ 3-fold loss of activity, while a reduced size (V581F) resulted in a ~ 2.5-fold decrease of activity towards linoleic acid (Fig. 4a). With the same mutations, similar differences in activities towards linolenic acid were observed in comparison to the wild type. Interestingly, the observed effects of the V581A/F mutations changed with increasing chain length of the fatty acid. Compared to C18-PUFAs, the V581F variant showed only a < 2-fold difference in activity towards C20-22:3(ω-3)-PUFAs eicosatrienoic acid and docosatrienoic acid (Fig. 4c, d). Furthermore, a ~ 2.5-fold increased activity against docosatrienoic acid was detected for the V581A variant, representing 50 % of the WT activity towards the natural substrate linoleic acid. Tightening the substrate tunnel receiving the carboxy-end of the PUFAs with the I393F variant, a ~ 3-fold decrease was observed against C18-PUFAs while C20- and C22-PUFAs were less affected by this mutation. CaeLox1 mutation W330L, which leads to a widening of the substrate tunnel, resulted in only ~ 15 % of the activity towards linoleic acid in comparison to the wild type LOX CaeLox1. Surprisingly in this mutant (W330L), the conversion of eicosatrienoic acid was increased by ~ 20 % compared to linoleic acid. However, the W330L variant showed a ~ 60 % reduction in the activity to docosatrienoic acid (Fig. 4c, d). Due to the increased specific activity of the W330L and V581A variants towards the C20-22:3(ω-3)-PUFAs, the double mutant W330L/V581A was created. This variant exhibited similar or even lower specific activities towards C18- as well as C20-22-PUFAs, compared to the single mutations V581A and W330L (Fig. 4a–d).

## Discussion

Both variants V581A and V581F showed similar activities to the fungal occurring PUFAs (linoleic acid and linolenic acid) compared to the wild type LOX, which suggests that neither increasing or decreasing space at that position leads to significantly higher activities but rather interfere with an efficient substrate binding of C18-PUFAs (Fig. 4a, b). The observed effects of the V581A/F mutations changed with increasing chain length, suggesting that alteration of space inside the tunnel seems to get relevant for longer chain PUFAs (Fig. 4c, d). Due to an additional C=C double bond in long chain PUFAs like C20(ω-3), which leads to lessened flexibility of the alkyl-chain, providing extra space seems to be beneficial. On the other hand, linoleic acid that harbors two C = C bonds resulting in increased flexibility of the alkyl-chain, seems to require a tighter substrate tunnel for an optimal substrate orientation. Although the increased chain length in comparison to the eicosatrienoic acid leads to a higher flexibility, the conformation of docosatrienoic acid in the substrate tunnel without a tryptophan might be unsuitable (Fig. 4c, d). Previous studies investigated the role of the amino acid residues W500 and W523, located in the substrate tunnel of LOX-1 from soybeans and a LOX from pea seeds (Hughes et al. 2001; Ruddat et al. 2004). Increasing space at position W500 of LOX-1 from soybeans revealed a decrease of activity towards linoleic acid and arachidonic acid (Ruddat et al. 2004). Furthermore, the W523A variant of a LOX from pea seeds showed no difference in activity towards linolenic acid but a ~ 6-fold decrease towards the longer arachidonic acid (Hughes et al. 2001). This is partly in accordance with our results, since we were able to show spacing at this position (corresponds to W330 in CaeLox1) plays a beneficial role for the C-20(ω-3)-PUFA eicosatrienoic acid. By combining the mutations V581A and W330L to the V581A/W330L variant, the beneficial effects of the single mutations for C-20-22(ω-3)-PUFAs seem to neutralize each other (Fig. 4c, d). This demonstrates the challenges in efficient protein engineering of lipoxygenases. Due to the long carbon chain of fatty acids, a large number of substrate conformations, their interactions with the residues of the tunnel as well as physicochemical effects have to be taken into account which makes a general prediction of important amino acid residues in the substrate tunnel difficult. Besides the mentioned studies, no other mutagenesis studies with long chain PUFAs are existent.

## Data Availability

All relevant data for this article are included within this manuscript. The GenBank accession number for the *LOX1* gene is MW013781.
